# The relationship between dementia staging scales, cognitive-behavioral scales and functionality in patients with cognitive impairment

**DOI:** 10.1371/journal.pone.0322572

**Published:** 2025-05-02

**Authors:** Fatma Ozge Kayhan Koçak, Emre Kumral

**Affiliations:** 1 Division of Geriatrics, Department of Internal Medicine, Health Sciences University Tepecik Training and Research Hospital, İzmir, Turkiye; 2 Department of Neurology, Faculty of Medicine, Ege University, İzmir, Turkiye; Andalusia Nursing Academy, INDONESIA

## Abstract

**Introduction:**

The aim of this study is to retrospectively evaluate the relationship between dementia stage, cognitive and behavioral scales, and functional status in patients with cognitive impairment.

**Methods:**

The medical records of patients over 50 years of age, who were followed up for cognitive impairment at the neurology outpatient clinic were retrospectively scanned between January 1990 and November 2022. The Clinical Dementia Rating (CDR), Global Deterioration Scale (GDS) and Functional Assessment Staging Test (FAST), The Mini Mental State Examination (MMSE) and the Alzheimer’s Disease Assessment Scale-Cognitive Subscore (ADAS-Cog) were recorded. The neuropsychiatric symptoms were evaluated by the Neuropsychiatric Inventory (NPI) and The Behavioral Pathology in Alzheimer’s Disease Rating Scale (BEHAVE-AD). Patients’ Instrumental Assessment of Daily Living (IADL) scores were recorded to assess functional capacity.

**Results:**

This study analyzed 871 patients with cognitive impairment, 69.8% of whom were having functional impairment. Alzheimer’s disease was the most common type of dementia (64.6%), with memory problems as the key symptom (65.6%). Neuropsychiatric symptoms such as hallucinations, delusion, and eating disturbances were significantly associated with disability (p < 0.001), while depression and anxiety were not. CDR scale was the strongest predictor of disability (OR: 4.9, AUC = 0.740), outperforming other dementia staging scales. Cognitive and behavioral scales like MMSE and NPI showed stronger correlations with functional impairment than with the dementia staging scales (-0.132 < r_s_ < 0.472, p < 0.001 and -0.284 < r_s_ < -0.357, p < 0.001, respectively).

**Conclusion:**

Our study demonstrated that both cognitive status and behavioral symptoms are critical in determining the level of functional impairment in cognitive impairments, but their contributions differ in magnitude and focus. As well as cognitive decline, neuropsychiatric symptoms may also need targeted management to reduce their impact on functionality. We need a practical tool that can be used in all stages of dementia, that does not overlook the impact of neuropsychological symptoms, and that can assess ADL according to the needs of patients and carers.

## Introduction

Cognitive impairment is a broad definition; it covers a wide range of definitions, from cognitive function not performing adequately for age to cognitive impairment in more than one domain of cognitive function. The incidence of dementia increases with age, with less than 5% of the population under the age of 75 being diagnosed with dementia, rising to 17% of people over the age of 80 [1]. Alzheimer’s disease (AD), the most common form of dementia, may affect one in 10 adults aged 65 and older and one in three of those aged 85 and older [2].

Dementia, one of the most widely used definitions of cognitive impairment, is diagnosed not only by assessing cognitive status but also by assessing functional and behavioral status. Cognitive impairment screening tests such as the Mini-Mental State Examination (MMSE) show the severity of dementia, but they only assess cognitive outcomes and are often influenced by education level. So, as cognitive screening tools only assess cognitive status and can be affected by education level, dementia staging scales such as the Clinical Dementia Rating (CDR) [3], Global Deterioration Scale (GDS) [4] and Functional Assessment Staging Test (FAST) [5] are used, which cover activities of daily living, behavioral disturbance and cognition and are not affected by patients’ education level. However, the GDS and CDR do not classify the severity of cognitive impairment in the same way; for example, early-stage dementia is a CDR score of 2, whereas the GDS stage is 3 [6]. The Functional Assessment Staging Test (FAST) is a GDS-derived staging test, where the last two stages of the GDS are staged in more detail. It can be questioned retrospectively and is of differential diagnostic importance in patient care [5].

Functional status can be impaired at different levels throughout cognitive impairment. Furthermore, since cognitive function can be influenced by IQ, years of education, and occupational status, disease burden does not necessarily predict the severity of clinical symptoms or the impairment in daily functioning [7]. While instrumental activities of daily living, such as paying bills, may attract attention in the early stages for someone with a high level of education, they cannot be valued in the early stages for someone with no education. So, although treatment is more beneficial in the early stages of dementia, it is generally started when the functional decline is reported by the patient and their family. From the time of diagnosis, functional status is therefore often monitored through activities of daily living.

It has been shown that there is a close relationship between functional status and cognitive performance, even when cognitive function is normal [8]. In particular, a close relationship has been observed between the visual spatial function and the speed of reaction and the cognitive function [9]. Impairments in domains that require more cognitive functionality, such as paying bills or using the telephone, than physical functionality, such as housekeeping or food preparation, may be more affected by cognitive status [9].

As functional impairment can be influenced by both the stage and type of cognitive impairment, CDR and GDS systems are often used in practice for staging cognitive impairment. Studies have shown that functional impairment in mild cognitive impairment is more likely to be associated with apathy and depression, while in moderate cognitive impairment it is more likely to be associated with a decline in cognitive functioning [10–12]. Neuropsychiatric symptoms may be a critical factor in the risk of cognitive and functional impairment [13]. It has also been found that the severity and frequency of neuropsychiatric symptoms have a negative impact on the functionality of people with dementia [14]. A recent study showed that Alzheimer’s disease causes more disability and severe neuropsychiatric symptoms than vascular dementia and MCI [15]. However, it has not yet been studied which of the staging systems is better at correlating with functionality and better at reflecting functional impairment. The aim of this study is to retrospectively evaluate the relationship between dementia stage as assessed by CDR, FAST and GDS and functional status as assessed by the Instrumental Assessment of Daily Living (IADL) in patients with cognitive impairment.

## Methods

Records of patients over 50 years of age who were followed up for cognitive impairment at the neurology outpatient clinic were retrospectively scanned between January 1990 and November 2022. Data from 1692 patients were retrospectively reviewed from the system between 27.03.2023 and 15.05.2023. Fifty-four under the age of 50, 44 with a zero IADL score, 614 with a zero CDR score, 109 missing data were excluded. Patients were included if their cognitive impairment had been staged using the CDR, FAST and GDS and if their IADL scores had been calculated. Patients’ age, sex, level of education, key symptom, type of dementia, duration of follow-up for cognitive impairment, age at onset of cognitive impairment, neurological examination, and neuropsychiatric symptoms such as depression, psychosis, anxiety, hallucinations, apathy, sleep disturbances, appetite changes, and current chronic diseases were recorded. Also, patients were classified for cognitive disorders by neurologist diagnosis and Diagnostic and Statistical Manual of Mental Disorders code.

We conducted a post hoc power analysis in G*Power 3.1 to determine whether the study sample size was adequate. A post hoc power analysis was conducted to assess the statistical power of our binary logistic regression model. Based on an odds ratio of 4.9, a baseline event rate of 70%, a sample size of 871 patients, the power to detect a significant association with a two-sided alpha of 0.5 was calculated to be 1.0.

## Cognitive and behavior assessment

The Standardized Mini Mental State Examination (S-MMSE) [16,17] and the Alzheimer’s Disease Assessment Scale-Cognitive Subscore (ADAS-Cog) [18,19] were used to assess cognitive function. TheS-MMSE is a tool that assesses orientation, memory, attention, calculation, recall, language, motor function and perception, and visuospatial ability, with a maximum score of 30. Permission to use S-MMSE was obtained from Molloy et al. The ADAS-Cog is a cognitive assessment test consisting of 11 sections that measure both cognitive function and mood and behavior. Higher scores indicate deterioration, with the highest score being 70.

The neuropsychiatric symptoms were evaluated by the Neuropsychiatric Inventory (NPI) [20,21] and The Behavioral Pathology in Alzheimer’s Disease Rating Scale (BEHAVE-AD) [22]. The NPI is a carer-based tool to assess 12 symptoms of dementia with a maximum score of 144. These include delusions, hallucinations, agitation/aggression, dysphoria/depression, anxiety, euphoria/excitement, apathy/indifference, disinhibition, irritability/lability, abnormal motor behavior, nocturnal behavior and appetite/eating disorders. Otherwise, the BEHAVE-AD is used to assess behavioral problems in dementia that are not due to the cognitive and functional impairments of dementia. It consists of 25 individual items that are rated on a 4-point scale of severity, ranging from 0 (not present) to 3 (very distressing to the patient or carer). Seven symptom clusters are assessed: paranoia, hallucinations, activity disturbance, aggression, circadian rhythm disturbance, affective disturbance, and anxiety and phobias. Higher scores indicate greater neuropsychiatric symptoms.

## Dementia Staging Scales

The Clinical Dementia Rating (CDR), Global Deterioration Scale (GDS) and Functional Assessment Staging Test (FAST) were recorded.

The Clinical Dementia Rating (CDR) [3,23] is derived from information gathered from detailed interviews with patients and their relatives, including history and clinical examination, and six domains are used to produce the overall CDR score: Memory, Orientation, Judgment and Problem Solving, Community Relations, Home and Hobbies, and Personal Care. Each domain is scored as follows: “0” (no impairment), “0.5” (questionable impairment), “1” (mild impairment), “2” (moderate impairment), and “3” (severe impairment). Scores for each of the six domains are collected based on the standard CDR protocol [3,23].

The Global Deterioration Scale (GDS) assesses the severity of primary degenerative dementias and describes the stages of cognitive decline, consisting of 7 stages [4]. Stages 4–7 are the stages of dementia. In stage 5, a person is no longer able to survive without help. By observing a person’s behavioral characteristics and comparing them with the GDS, carers can get a rough idea of where a person is in the disease process.

The Functional Assessment Staging Test (FAST) is a functional staging system designed to assess patients at different stages of dementia [5]. There are 7 FAST stages: 1 for normal adults, 2 for subjective cognitive impairment, 3 for mild cognitive impairment, 4 for mild dementia, 5 for moderate dementia, 6 for moderately severe dementia, and 7 for severe dementia. Stages 6 and 7 are further subdivided into sub-stages, with 6a to 6e representing moderate dementia, 7a to 7c representing severe dementia, and 7d to 7f representing very severe sub-stages. Studies have shown that FAST is a reliable and valid assessment technique for assessing functional impairment throughout the course of the disease, particularly in Alzheimer’s dementia, and highlights a characteristic progressive functional decline.

## Functional assessment

Patients’ IADL scores were recorded to assess functional capacity. The Lawton-Brody IADL requires complex planning and thinking, such as managing medications, paying bills, and using the telephone [24,25]. It measures eight domains and can be administered over 10–15 minutes and may provide an early warning of functional decline or signal the need for further evaluation. The maximum score is 8, with higher scores indicating greater independence in functioning. Patients were classified as “dependent” according to the IADLs scale if the score was < 8.

## Statistical analysis

Normality was assessed using the Kolmogorov-Smirnov test. Quantitative variables showing normal distribution were expressed as mean ± standard deviation, and those not showing normal distribution were expressed as median and interquartile range values. Chi-square (x²) test and Fisher’s exact test were used in the analysis of categorical variables, and these were expressed as frequency and percentage. T-test and Mann-Whitney U test were used in the analysis of quantitative variables. For correlation analysis, Pearson analysis was used for non-normally distributed data and Spearman analysis was used for normally distributed data. Cronbach’s alpha as an internal consistency reliability was calculated for the CDR. To confirm the validity of the FAST and GDS, Spearman correlation coefficients were calculated between them and the CDR. Contingency tables, logistics regression models, multivariate analysis and ROC curve statistical analysis were done to evaluate the prognostic efficacy of the scales and neuropsychiatric symptoms. *P *< 0.05 was considered statistically significant. Data analyses were performed using SPSS version 25.0 for Windows.

## Ethical statement

Verbal informed consent was obtained from all participants by a systematic and standardized process used in the neurology outpatient clinic where the study was performed. Participants or their legal guardians, when appropriate, were informed that their medical information may be used for research purposes. If they disagreed, they informed the physician taking care of them and a note was recorded in their chart. No refusal was recorded for this study. All patients’ records/information were anonymized and de-identified prior to analysis. As this is a retrospective study, consent to participate was given by the Department of Neurology and the Ethical Committee for Medical Research at Ege University. The research protocol was conducted in accordance with the Helsinki declaration and was approved by Ege University Medical Research Ethical Committee (Ethics committee decision no:23-2T/40 date: 15.02.2023).

## Results

**Fig 1:**
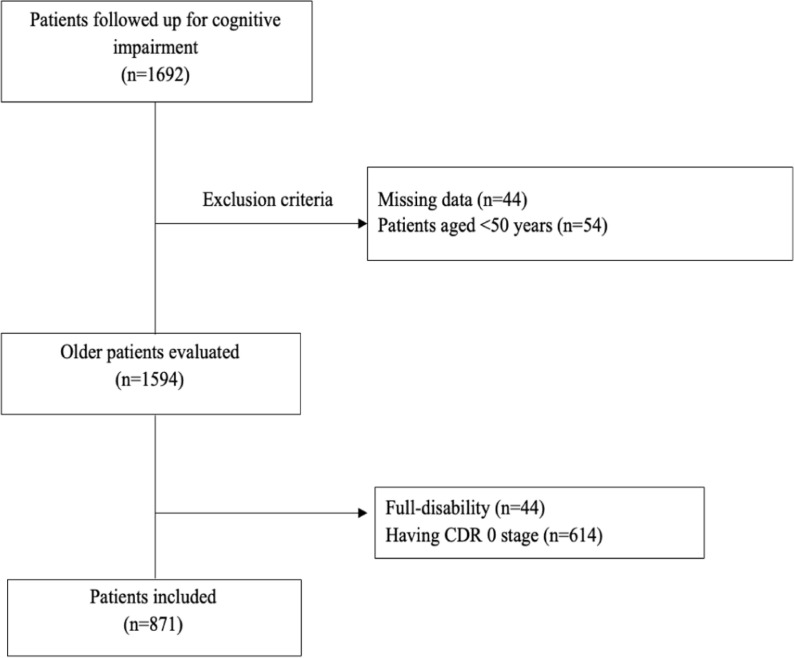
Flowchart of the study.

**Fig 2:**
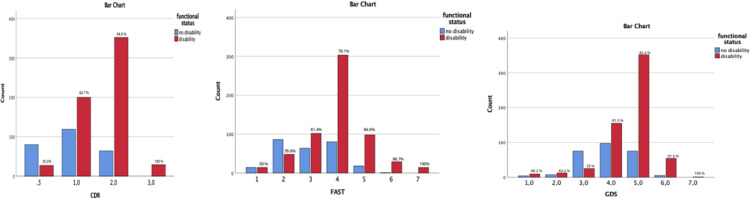
The bar chart of prevalence of disability in terms of dementia stage scales. The prevalence of disability is shown at the top of each bar chart. CDR; The Clinical Dementia Rating, FAST; Functional Assessment Staging Test, GDS; Global Deterioration Scale.

A total of 871 patients were included, of whom 696 (80%) were aged 65 years or older. The inclusion and exclusion criteria are explained in detail in **Fig 1**. There were 455 (52.2%) female patients. The mean NPI, BEHAVE-AD, ADAS-Cog and MMSE scores are 80 [45], 45 [28], 40 [20] and 20 [28] respectively. According to IADL, 608 (69.8%) patients was classified as functional impairment. 93 patients had mild cognitive impairment (MCI) according to physician diagnosis. Memory problems were the most common key symptom, while the most common type of dementia is Alzheimer disease. Baseline characteristics of study was shown at **Table 1**. According to stage of dementia, the prevalence of disability was shown as bar chart in **Fig 2**.

**Table 1:** The baseline characteristics of study.

Variables	All
Age, year, mean±SD	71.4 ± 8.3
Female, n (%)	455 (52.2)
Education year, mean±SD	6.6 ± 3.7
Key symptom	
Memory problems, n (%)	550 (65.6)
Memory and Behavioral problems, n (%)	202 (24.1)
Behavioral problems, n (%)	20 (2.4)
Dementia type	
Alzheimer, n (%)	551 (64.6)
MCI, n (%)	93 (10.9)
Frontotemporal dementia, n (%)	67 (7.9)
Mixt dementia, n (%)	56 (6.6)
Vascular dementia, n (%)	33 (3.9)
Follow-up duration, month, mean±SD	39.9 ± 64.1
Age at onset, year, mean±SD	68 ± 9.6

MCI; mild cognitive impairment

**Fig 3:**
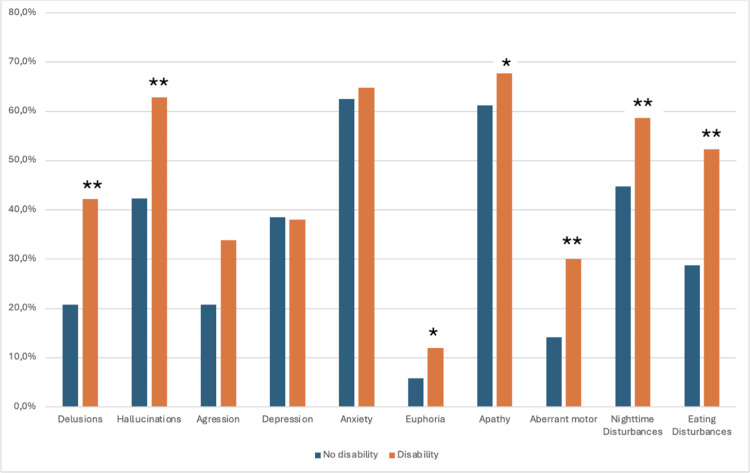
Prevalence of neuropsychiatric symptoms in terms of disability. Neuropsychiatric symptoms were more common in dementia patients with disabilities than in those without disabilities. Significant difference between groups; **p < 0.001, * p < 0.05.

In terms of each symptom, apathy was the most frequent neuropsychiatric symptom observed in patients with disability, reaching 71%. It was followed by anxiety (65%), hallucination (64%), and nightmare disturbance (59%). The frequency of all these symptoms, excluding aggression, depression and anxiety, was significantly higher in patients with disability than in those without disability (35.5 > x^2^ > 7.5, p < 0.001) (**Fig 3**). All neuropsychiatric symptoms, excluding depression and anxiety, were associated with disability in univariate regression analysis. Delusion, hallucination and eating disturbances was significantly associated with disability in univariate and multivariate analysis. The regression analysis of the study was shown at **Table 2** and **Table 3**.

**Table 2:** Logistic regression analysis of disability and neuropsychiatric symptoms.

	Disability					
Variables	Univariate			Multivariate		
	OR	Cl (95%)	P	OR	Cl (95%)	P
Delusion	0.36	0.25 - 0.51	**<0.001**	0.57	0.38 - 0.84	**0.004**
Hallucination	0.42	0.31- 0.57	**<0.001**	0.64	00.44–0.93	**0.02**
Aggression	0.51	0.36 - 0.73	**<0.001**	0.73	0.50–1.07	0.11
Depression	1.02	0.75 -1.38	0.9	0.92	0.66–1.27	0.59
Anxiety	0.91	0.67–1.23	0.5	1.3	0.90–1.83	0.17
Euphoria	0.45	0.25–0.81	**0.007**	0.71	0.38–1.32	0.28
Apathy	0.65	0.48 - 0.88	**0.006**	0.92	0.64–1.33	0.66
Aberrant motor	0.39	0.26 - 0.57	**<0.001**	0.79	0.49–1.26	0.32
Nighttime disturbances	0.57	0.42 - 0.78	**<0.001**	0.83	0.59–1.16	0.27
Eating disturbances	0.37	0.27–0.51	**<0.001**	0.59	0.40–0.87	**0.008**

OR; Odds ratio, CI; Confidence interval,

**Table 3:** Regression analysis showing the association of IADL scores (dependent variable) with the dementia staging scales, cognitive and behavioral scales (independent variable, separated model for them) (n = 871).

	*Linear regression* ^*^			*Univariate logistic regression* ^†^		
	^ß^	*95% CI*	*P-value*	OR	*95% CI*	*P-value*
Dementia staging scales						
FAST	-0.54	7.53;8.33	**< 0.001**	2.1	1.82;2.45	**< 0.001**
GDS	-0.38	7.06;8.27	**< 0.001**	2.2	1.84;2.58	**< 0.001**
CDR	-0.57	6.49;7.14	**< 0.001**	4.9	3.73;6.54	**< 0.001**
Cognitive and behavioral scales						
NPI	-0.019	7.42;7.98	**< 0.001**	1.0	1.04;1.05	**< 0.001**
BEHAVE-AD	-0.018	6.76;7.35	**< 0.001**	1.1	1.04;1.06	**< 0.001**
ADAS-COG	0.017	5.03;5.68	**< 0.001**	1.0	1.02;1.04	**< 0.001**
MMSE	0.080	3.95;4.94	**< 0.001**	0.7	0.67;0.75	**< 0.001**

ß ; Coefficient of regression beta, OR; Odds ratio, CI; Confidence interval,

* *AIDL score used as a continuous dependent variable*

† *Disability used as a discontinuous variable defined as AIDL score less than 8*

The CDR score has been accepted as the gold standard for staging cognitive impairment. The Cronbach’s alpha of the CDR was between 0.90–0.91. This indicates that the level of reliability is acceptable. The FAST and GDS showed a moderate correlation with the CDR (r_s_:.400 p < 0.001 and r_s_:.630 p < 0.001 respectively).

**Fig 4:**
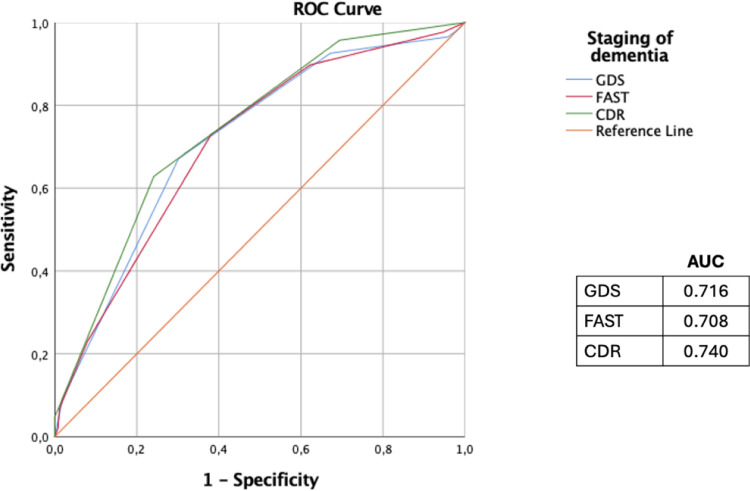
ROC curves of the different scales of staging of dementia to analyze the strength of association with disability. CDR; The Clinical Dementia Rating, FAST; Functional Assessment Staging Test, GDS; Global Deterioration Scale, AUC; area under curve.

Overall, within all participants, the IADL scale was more strongly correlated with the cognitive and behavioral scales, except ADAS-Cog, (-0.350 < r_s_ < 0.472, p < 0.001) than with the dementia staging scales (-0.284 < r_s_ < -0.357, p < 0.001). The correlation analysis was shown in **Table 4**. The ROC curve analysis highlighted the CDR a greater area under the curve (AUC = 0.740) than the others, to relate dementia with disability. AUC of CDR, FAST, GDS are shown in **Fig 4**.

**Table 4:** Correlations (Spearman’s Rho) for functional impairment with variables.

Variables	N	Median	IQR	r_s_	P value
**Dementia staging scales**					
FAST	871	4	1	-0.357	**<0.001**
GDS	871	5	1	-0.284	**<0.001**
CDR	871	2	1	-0.304	**<0.001**
**Cognitive and behavioral scales**					
NPI	800	80	45	-0.435	**<0.001**
BEHAVE-AD	803	45	28	-0.350	**<0.001**
ADAS-COG	845	40	20	-0.132	**<0.001**
MMSE	861	20	6	0.472	**<0.001**
**Neuropsychiatric symptoms**					
Delusion	807	NA	NA	0.201	**<0.001**
Hallucination	808	NA	NA	0.183	**<0.001**
Aggression	813	NA	NA	0.157	**<0.001**
Depression	805	NA	NA	-0.033	0.35
Anxiety	806	NA	NA	-0.030	0.39
Euphoria	798	NA	NA	0.149	**<0.001**
Apathy	805	NA	NA	0.034	0.33
Aberrant motor	814	NA	NA	0.241	**<0.001**
Nighttime disturbances	800	NA	NA	0.105	**0.003**
Eating disturbances	837	NA	NA	0.247	**<0.001**

IQR, Interquartile Range, NA; not applicable, r_S_; Spearman’s Rho

Coefficient of regression beta, odds ratio, hazard ratio and P-value significant (i.e., < 0.05) indicated in bold.

## Discussion

This study evaluated the relationship between dementia staging scales, cognitive and behavioral scales and functionality in patients with cognitive impairment. Our study indicates that among dementia staging scales, CDR demonstrated the strongest predictive value for disability in patients with cognitive impairment. Also, functional impairment measured by IADL is significantly associated with neuropsychiatric symptoms. We showed that having delusions, hallucinations or eating disorders increased the risk of disability by almost 50%.

Among dementia staging tools, the CDR has the best predictive accuracy for functional disability in our study, making it the preferred scale for assessing functional decline in clinical practice. CDR stages dementia by focusing on cognitive domains rather than functionality, compared with FAST and GDS. A recent cross-sectional study found that the eCDR can be used to predict cognitive and functional decline in older patients with dementia [26]. In a single-center observational study, cognitive function was the only factor influencing functional disability in patients with severe dementia at 6 months’ follow-up [27]. In a cohort study followed for 8 years, it was found that the early diagnosis of dementia increased as the severity of cognitive symptoms increased [28].

Impairments in language and practical skills can seriously affect instrumental ADL [29]. Cognitive scales like MMSE and behavioral scales like NPI better predict functional impairment compared to dementia staging scales, although staging scales like CDR remain highly significant in our study. These findings highlight the importance of neuropsychiatric symptoms in the functional status of patients with cognitive impairment. A similar study showed that behavioral and psychological symptoms assessed by the NPI and functional disability were also associated with poorer quality of life [30]. Unlike our study, Tanaka et al found that behavioral and psychological symptoms assessed by the NPI were not found to be associated with functional disability [27].

We found that neuropsychiatric symptoms such as delusions, hallucinations, and eating disturbances have a significant, independent negative impact on functional independence in dementia patients. Hallucinations are more common with the progression of dementia [31]. Apathy has also been shown to be the most predictive of functional impairment in frontotemporal dementia [32–34]. Salech et al showed that apathy was strongly associated with functional impairment in patients with behavioral variant frontotemporal dementia [34].

Interestingly, depression and anxiety, which are common in dementia, did not show significant associations with functional disability in the multivariate analysis. This suggests that these symptoms alone may not directly contribute to functional decline when adjusted for other variables. Similarly, the functional impairment due to hospitalisation was found to be reversible in patients with depression compared with dementia [35]. On the other hand, depressive mood was found to be negatively correlated with basic ADL, but not with complex ADL [29].

People with dementia have a worsening functional status in the long term [36]. In particular, older nursing home residents with cognitive impairment are more likely to experience functional impairment and, in particular, difficulties with eating, walking and bed mobility are also risk factors for mortality [37]. Patients with cognitive concerns without a diagnosis of dementia had poorer recovery and greater disability during hospitalization than those with a diagnosis of dementia, according to a study conducted in a geriatric inpatient unit [38]. In contrast, a prospective cohort study with a 3-month follow-up showed that older patients with dementia had less recovery of functional improvement after discharge than those without dementia [35]. In the comparison of these two studies, the early diagnosis of dementia is as important for the patient’s functioning as is the presence of dementia.

An eighteen-month follow-up study of people with mild Alzheimer’s disease found that cognitive impairment was more prominent than functional impairment in the early stages of the disease. However, there was an increase in the correlation between the two over time, and the functional improvement with treatment was mainly due to cognitive improvement [39]. Similarly, another observational study found that a lower level of functional impairment was associated with being unaware of a diagnosis of dementia in the older patients [40]. Also, Connors et al. showed that changes in functional impairment and severity of dementia over time were predictive of mortality, regardless of baseline levels [41]. It seems that in people with dementia, functional impairment and cognitive impairment are two inseparable parts.

Our study has some limitations. Tools like the CDR, FAST, and GDS, as well as neuropsychiatric symptom scales, rely on clinical judgment or caregiver reports. So, subjective biases in ratings could influence the results, especially if caregivers underreport or overreport symptoms. Our study is having limited generalizability. Excluding patients under 50 and those with zero IADL scores may introduce a selection bias; even patients with early dementia may still have some functional impairment that is not captured by standard IADL scales. The findings may not apply to all dementia populations, especially those with differing cultural, socioeconomic, or healthcare contexts. There are also many factors (e.g., comorbidities, medication, or cognitive training interventions) that influence functional status in cognitive disorders. The lack of assessment of some known independent factors is a limitation of our study. As IADL was not be perfectly linear and ADAS-COG and MMSE may be correlated with other factors influencing IADL scores, the result of linear regression could also be misleading. Our study did not assess the progression of symptoms or functional disability. This limits our understanding of how specific neuropsychiatric symptoms influence disability trajectories.

## Conclusions

In this century of increasing life expectancy, living independently has become the most important health strategy. In particular, quality of life and quality of care for people with cognitive impairment is closely related to their functional status. As well as cognitive decline, neuropsychiatric symptoms may also need targeted management to reduce their impact on functionality. Longitudinal studies are essential to establish the benefits of interventions and the effect of treatment and symptoms on disease progression over time. Further investigation into the impact of other neuropsychiatric and physical comorbidities is needed to gain a comprehensive understanding of their roles. We need a practical scale that can be used in all stages of dementia, that does not overlook the impact of neuropsychological symptoms, and that can assess ADL according to the needs of patients and carers.

## Abbreviations:

CDR: The Clinical Dementia Rating
GDS: Global Deterioration Scale
FAST: Functional Assessment Staging Test
MMSE: The Mini Mental State Examination
ADAS-Cog: the Alzheimer’s Disease Assessment Scale-Cognitive Subscore
NPI: Neuropsychiatric Inventory
BEHAVE-AD: The Behavioral Pathology in Alzheimer’s Disease Rating Scale
AD: Alzheimer’s disease
IADL: the Instrumental Assessment of Daily Living
AUC: area under curve
IQR: Interquartile Range
OR: Odds ratio
CI: Confidence interval
MCI: mild cognitive impairment.
